# AI-enhanced entrepreneurship education in vocational colleges: the roles of strategic engagement, digital literacy, and perceived fairness

**DOI:** 10.3389/fpsyg.2026.1826836

**Published:** 2026-05-13

**Authors:** Yiwu Tang, Xinyi Qi, Yuchen Gao, Peiqing Sun

**Affiliations:** 1 Department of Basic Teaching, Chuzhou Polytechnic, Chuzhou, China; 2 School of Management, Chuzhou Polytechnic, Chuzhou, China; 3 School of Chemistry and Chemical Engineering, Nanjing University, Nanjing, China

**Keywords:** artificial intelligence, educational psychology, entrepreneurship education, student engagement, vocational education

## Abstract

**Introduction:**

Generative artificial intelligence (AI) is increasingly entering higher education, yet its educational-psychological implications for vocational entrepreneurship education remain underexplored.

**Methods:**

This quasi-experimental mixed-methods study examined whether AI-enhanced entrepreneurship instruction improves learning outcomes in vocational colleges and how strategic engagement, digital literacy, and perceived fairness shape those outcomes. Participants were 360 students from 12 vocational colleges (180 AI-enhanced; 180 comparison), together with instructors and industry mentors who contributed implementation and assessment perspectives. Quantitative data included pre/post business plan scores, self-reported entrepreneurial competence, project outcome, and digital literacy assessments; qualitative data came from focus groups, instructor interviews, and mentor feedback.

**Results:**

Students in the AI-enhanced condition showed larger gains in business plan performance than controls [22.4% vs. 15.1%; mean difference = 7.3 percentage points, 95% CI (4.3, 10.3), Cohen’s d = 0.50]. Strategic Prompters outperformed Passive Consumers and Minimal Users, and students with stronger digital literacy benefited more from AI-supported learning. Instructors reported continuing concerns about fairness, transparency, authorship, and bias, while students from lower socio-economic backgrounds reported smaller perceived equity gains.

**Discussion:**

The findings suggest that AI can support entrepreneurship learning in vocational colleges when it is embedded within psychologically informed pedagogy emphasizing self-regulation, critical evaluation, and transparent assessment.

## 1 Introduction

Entrepreneurship education has become an increasingly important component of vocational higher education because vocational colleges are expected to prepare graduates who can respond to technological change, labor-market uncertainty, and the practical demands of industry and self-employment (Nabi et al., 2017; Handayati et al., 2020; Li and Islam, 2021; Martinez and Pelaez-Higuera, 2024). In this setting, entrepreneurship education is not simply a matter of transmitting business knowledge. It also aims to cultivate opportunity recognition, creative problem-solving, collaborative judgment, persistence under uncertainty, and confidence in turning ideas into viable action. These are educational-psychological concerns because they are rooted in how students engage, regulate learning, and develop beliefs about competence and agency (Ryan and Deci, 2000; Zimmerman, 2002; Fredricks et al., 2004; Panadero, 2017).

Despite its importance, entrepreneurship education in vocational settings continues to face persistent instructional constraints. Traditional approaches often rely on teacher explanation, fixed cases, delayed feedback, and limited opportunities for iterative revision. Yet entrepreneurship learning is inherently dynamic. Students are typically asked to generate ideas, assess market feasibility, refine value propositions, and justify decisions under ambiguity. When adaptive support is weak, students may complete tasks procedurally rather than strategically, which restricts deeper learning and weakens the development of self-regulated, motivated participation (Boekaerts and Corno, 2005; Nicol and Macfarlane-Dick, 2006).

Recent advances in generative AI have introduced new possibilities for addressing these instructional challenges. Large language models and AI-supported feedback systems can provide rapid idea generation, prompt-based coaching, scenario simulation, and iterative revision support (Zawacki-Richter et al., 2019; Dwivedi et al., 2023; Kasneci et al., 2023; Tlili et al., 2023). More recent syntheses suggest that generative AI can support self-directed and self-regulated learning, but that its benefits are conditional rather than automatic (Mai et al., 2024; Lan and Zhou, 2025; Xia et al., 2026). In other words, AI does not improve learning simply by being available; it changes the conditions under which students plan, monitor, and evaluate their work.

Three constructs are especially important for understanding these conditions. In the present study, strategic engagement refers to goal-directed, evaluative, and revision-oriented use of AI in which students compare alternatives, refine prompts, and judge the quality of AI output rather than accepting it uncritically. This definition is grounded in educational psychology research on cognitive engagement and self-regulated learning (Zimmerman, 2002; Fredricks et al., 2004; Panadero, 2017). Digital literacy refers to the ability to locate, evaluate, interpret, and integrate digital and AI-generated information into meaningful academic work (Ng, 2012; van Laar et al., 2017; Zhao et al., 2024). Perceived fairness refers to students’ and instructors’ judgments that AI-assisted learning and assessment are transparent, consistent, and equitable with respect to authorship, access, and evaluative standards (Selwyn, 2022; Cotton et al., 2024; Kofinas et al., 2025).

These issues are especially salient in vocational colleges, where students often enter with uneven prior digital experience and varying access to support. Emerging work on the ‘GenAI divide’ warns that equal access to tools does not necessarily translate into equal educational benefit, because learners differ in capabilities and in their capacity to convert access into advantage (Beckman et al., 2025). Recent higher education studies similarly suggest that AI-related self-learning benefits are shaped by student characteristics, including learning difficulty status, familiarity with AI, and prompting experience (Saleh and Elsayary, 2026).

**Figure 1:**
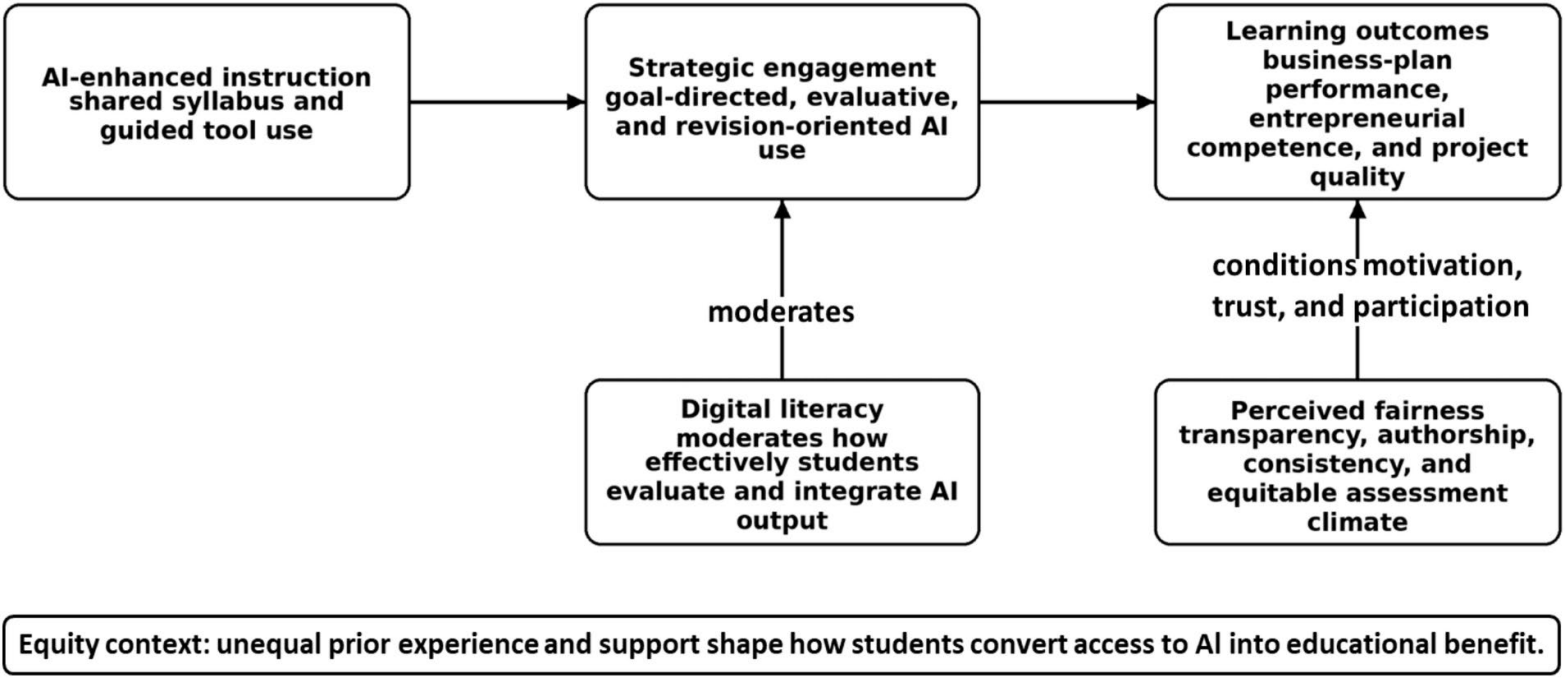
Conceptual framework linking AI-enhanced instruction to learning outcomes through strategic engagement, moderated by digital literacy and conditioned by perceived fairness and equity context.

Building on these developments, we conceptualized AI-enhanced entrepreneurship education as a psychologically mediated learning ecology. Rather than treating AI as a stand-alone technical intervention, the study examined how instructional design, student engagement, digital literacy, and fairness perceptions jointly shaped learning. Figure 1 summarizes the conceptual framework, and Table 1 clarifies the focal constructs and their operationalization in the study.

**Table 1:** Conceptual definitions and study operationalization of the focal constructs.

Construct	Conceptual definition	Study operationalization
Strategic engagement	Goal-directed, evaluative, and revision-oriented use of AI.	AI-use records, brief self-reports, and instructor observations; classified as Strategic Prompters, Passive Consumers, or Minimal Users.
Digital literacy	Ability to evaluate and integrate digital and AI-generated information.	Structured assessment aligned with entrepreneurship tasks and informed by prior digital literacy frameworks.
Perceived fairness	Judgment that AI-assisted learning and assessment are transparent, consistent, and equitable.	Student and instructor reports on transparency, authorship, bias, consistency of assessment, and unequal benefit.

The study addressed four research questions: (1) Does AI-enhanced entrepreneurship instruction improve business-plan performance and entrepreneurial competence relative to traditional instruction? (2) How do students differ in their patterns of engagement with AI tools, and how are these patterns associated with project outcomes? (3) Does digital literacy moderate the relationship between AI-enhanced instruction and learning gains? (4) How do students and instructors perceive the fairness and equity implications of AI-assisted learning and assessment?

In line with these questions, four hypotheses guided the quantitative analysis. H1: Students in the AI-enhanced condition will show greater gains in business-plan performance and entrepreneurial competence than students in the comparison condition. H2: Within the AI-enhanced condition, students showing strategic engagement will achieve stronger project outcomes than passive or minimal users. H3: Digital literacy will positively moderate the relationship between AI-enhanced instruction and learning gains. H4: Perceived fairness and equity will be more fragile in relation to AI-assisted work, especially for students with fewer prior digital resources or support.

By addressing these questions, the study aims to contribute to educational psychology in two ways. First, it extends research on AI in education beyond broad claims of effectiveness by identifying the learner processes through which AI may support or constrain learning. Second, it brings entrepreneurship education into dialogue with current debates on self-regulated learning, fairness, and educational equity in AI-supported environments.

## 2 Materials and methods

### 2.1 Design

This study employed a quasi-experimental mixed-methods design. The quantitative component compared learning outcomes between an AI-enhanced instructional condition and a traditional instruction condition. The qualitative component examined how students and instructors experienced AI-supported learning, including differences in AI use, concerns about fairness, and perceptions of unequal benefit. This design was selected because it allowed the study to examine both outcome differences and the educational-psychological processes that may help explain those differences.

Because intact classes rather than individual students were assigned to conditions, the study was quasi-experimental rather than randomized. Whenever feasible, participating colleges contributed parallel classes within the same entrepreneurship module so that the AI-enhanced and comparison conditions were matched at the course level. The analytic goal was therefore to estimate practically meaningful differences under authentic instructional conditions while acknowledging the limits of causal inference.

### 2.2 Participants and setting

Participants were 360 students enrolled in entrepreneurship-related courses across 12 vocational colleges. Of these, 180 students participated in the AI-enhanced condition and 180 in the comparison condition. Students were eligible if they were enrolled in the focal course during the study semester, had not previously completed the same entrepreneurship module, and provided informed consent for their coursework and survey responses to be analyzed for research purposes.

Participating colleges were recruited on the basis of willingness to implement a common entrepreneurship teaching sequence, availability of the required digital infrastructure, and instructor agreement to follow the shared assessment procedures. Instructors were included if they were responsible for the focal course and participated in the study briefing on curriculum alignment, assessment expectations, and responsible AI use. Across institutions, baseline course records indicated that the two student groups were broadly comparable in age, gender distribution, and socio-economic composition.

In addition to student participants, instructors provided feedback on implementation, assessment, and ethical concerns, and industry mentors contributed evaluations of the authenticity and practical quality of student business plans in order to strengthen the ecological validity of project assessment.

### 2.3 Instructional conditions

Both conditions followed the same entrepreneurship curriculum, centered on opportunity identification, market analysis, value proposition design, business-model development, and pitch preparation. The teaching sequence, major assignments, scoring rubrics, and mentor evaluation criteria were standardized across participating classes through a shared syllabus and instructor briefing materials.

Students in the AI-enhanced condition used institutionally permitted generative AI tools, AI-supported business-simulation activities, and real-time feedback systems during entrepreneurship tasks. AI was used for brainstorming, market analysis, business-plan refinement, and pitch preparation. At the start of the module, students received structured guidance on responsible AI use, including prompt design, output verification, citation/authorship expectations, and the need to justify decisions independently. Instructors also provided example prompts and revision routines, but students were encouraged to adapt these prompts rather than follow them mechanically.

Students in the comparison condition completed the same curriculum without AI support. They received teacher guidance, standard course materials, peer collaboration opportunities, and conventional feedback on business-planning tasks. This design made it possible to compare AI-enhanced instruction against a pedagogically comparable non-AI baseline rather than against an unrelated instructional format.

### 2.4 Measures and data sources

Business-plan performance was assessed using pre- and post-intervention tasks scored with a common rubric covering coherence, feasibility, market relevance, and integration of entrepreneurial reasoning. To reduce expectancy bias, submissions were anonymized before scoring wherever feasible and were evaluated using the same rubric across conditions.

Entrepreneurial competence was measured through student self-report indicators of opportunity recognition and business model innovation. These items were aligned with the learning objectives of the shared curriculum and were framed as perceived capability gains rather than as direct behavioral tests.

Strategic engagement was operationalized through students’ patterns of AI use in the AI-enhanced condition. Drawing on weekly task records, brief self-reports, and instructor observations, students were classified into three dominant engagement profiles: Strategic Prompters, Passive Consumers, and Minimal Users. Strategic Prompters were characterized by prompt iteration, comparison of outputs, and revision decisions justified against course criteria; Passive Consumers tended to accept AI output with limited evaluation; and Minimal Users engaged only weakly with the tools.

Digital literacy was assessed through a structured evaluation of students’ ability to locate, evaluate, interpret, and apply digital and AI-generated information in academic tasks. The assessment drew conceptually on prior digital competence and digital literacy frameworks (Ng, 2012; van Laar et al., 2017; Zhao et al., 2024) while being adapted to the entrepreneurship tasks used in the present study.

Perceived fairness and equity were examined through student reports and instructor responses concerning transparency, authorship, bias, consistency of assessment, and whether AI-supported learning advantages were distributed evenly across student groups. Industry mentor evaluations were used to complement course-based scoring and to assess the practical authenticity of the final projects.

### 2.5 Instrument development and construct validity

Construct development was guided by the educational psychology literature reviewed in the Introduction. Strategic engagement, digital literacy, and perceived fairness were defined *a priori* and then linked to concrete indicators appropriate for entrepreneurship instruction. Item wording and rubric language were reviewed for clarity and alignment with course tasks before full implementation, and the final instruments combined literature-informed constructs with task-specific adaptation to the vocational context.

Because the study included both performance-based and self-report measures, construct validity was addressed through design triangulation rather than reliance on a single instrument. For example, entrepreneurial competence was assessed through self-report indicators, but project quality was also evaluated through rubric-based performance scores and mentor feedback; similarly, fairness was examined through both student and instructor perspectives. This multi-source strategy was intended to strengthen interpretive confidence in the findings.

### 2.6 Qualitative component and thematic analysis

The qualitative component included student focus groups, instructor interviews, and open-ended implementation notes. Student groups were purposively sampled to capture variation in AI engagement profiles, achievement, and socio-economic background. Instructor interviews focused on preparedness to assess AI-assisted work, perceptions of authorship and transparency, and concerns about bias or uneven benefit.

Qualitative data were analyzed thematically using a combined deductive-inductive approach. An initial coding frame was derived from the study constructs (strategic engagement, digital literacy, fairness, equity), after which the researchers iteratively refined codes to capture emergent themes such as dependence on AI output, uncertainty in judging authentic reasoning, and unequal benefit despite equal access. Coding decisions were compared across the research team and discrepancies were resolved through discussion to improve analytic consistency.

### 2.7 Data analysis

Quantitative analyses included descriptive statistics, independent-samples t-tests, and multiple regression analyses. Group comparisons examined whether the AI-enhanced condition produced stronger gains than the comparison condition. In addition to *p* values, effect sizes and 95% confidence intervals were reported for the main inferential comparisons in order to improve interpretability.

Regression analyses were used to test the association between AI engagement and project outcomes and to examine the moderating role of digital literacy. Moderation was modeled through interaction terms and further interpreted by comparing learning gains across high, medium, and low digital literacy groups. Qualitative findings were then used to contextualize the quantitative patterns and identify plausible mechanisms behind heterogeneous effects.

### 2.8 Ethics statement

All procedures involving human participants were conducted in accordance with institutional ethical requirements and the principles of informed consent, confidentiality, and anonymity. Students were informed that AI tools were to be used as learning supports rather than as substitutes for independent judgment. Participation in surveys and interviews was voluntary, and research analyses were conducted on de-identified data.

During the intervention, instructors discussed responsible AI use, authorship, transparency, and fairness. The study therefore involved human participants and was reviewed according to the relevant institutional procedures at the participating colleges.

## 3 Results

### 3.1 Effects of AI-enhanced instruction on business plan performance

**Figure 2:**
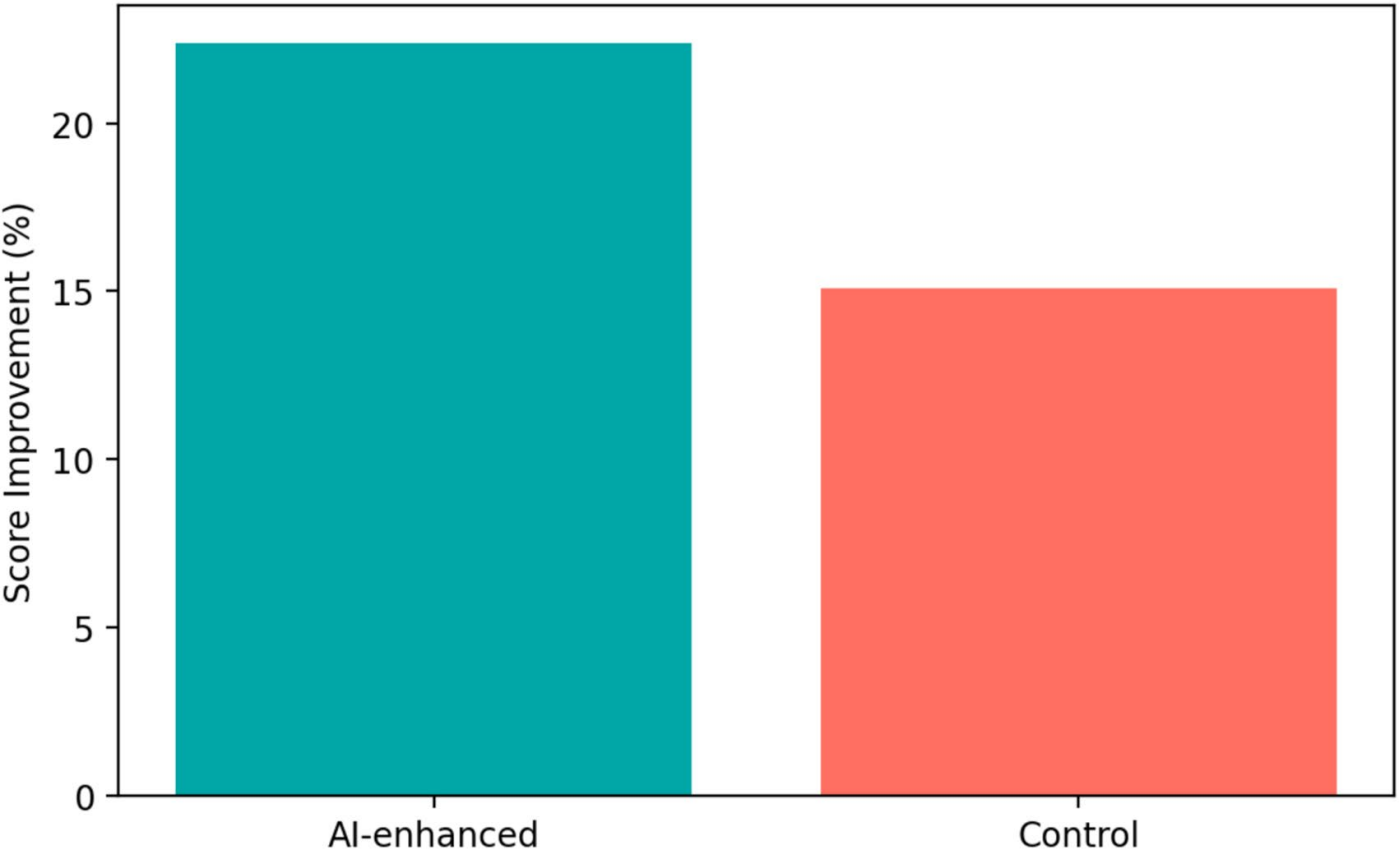
Business plan score improvement (%) in the AI-enhanced vs. traditional instruction groups from pre- to post-intervention.

The integration of AI tools into the entrepreneurship curriculum was associated with significantly stronger gains in business-plan performance. As shown in Figure 2, the AI-enhanced group demonstrated an average improvement of 22.4% in business plan scores, compared with 15.1% in the comparison group. An independent-samples *t*-test showed that this difference was statistically significant, *t* (358) = 4.73, *p* < 0.01. The mean difference was 7.3 percentage points, with a 95% confidence interval of [4.3, 10.3], corresponding to a medium effect size (Cohen’s d = 0.50).

From an educational psychology perspective, this result suggests that AI-supported environments may enhance performance when they provide timely scaffolding during complex and cognitively demanding tasks. In entrepreneurship learning, rapid feedback and iterative revision opportunities appear to help students refine ideas and produce more coherent project outputs.

### 3.2 Self-reported entrepreneurial skill development

**Figure 3:**
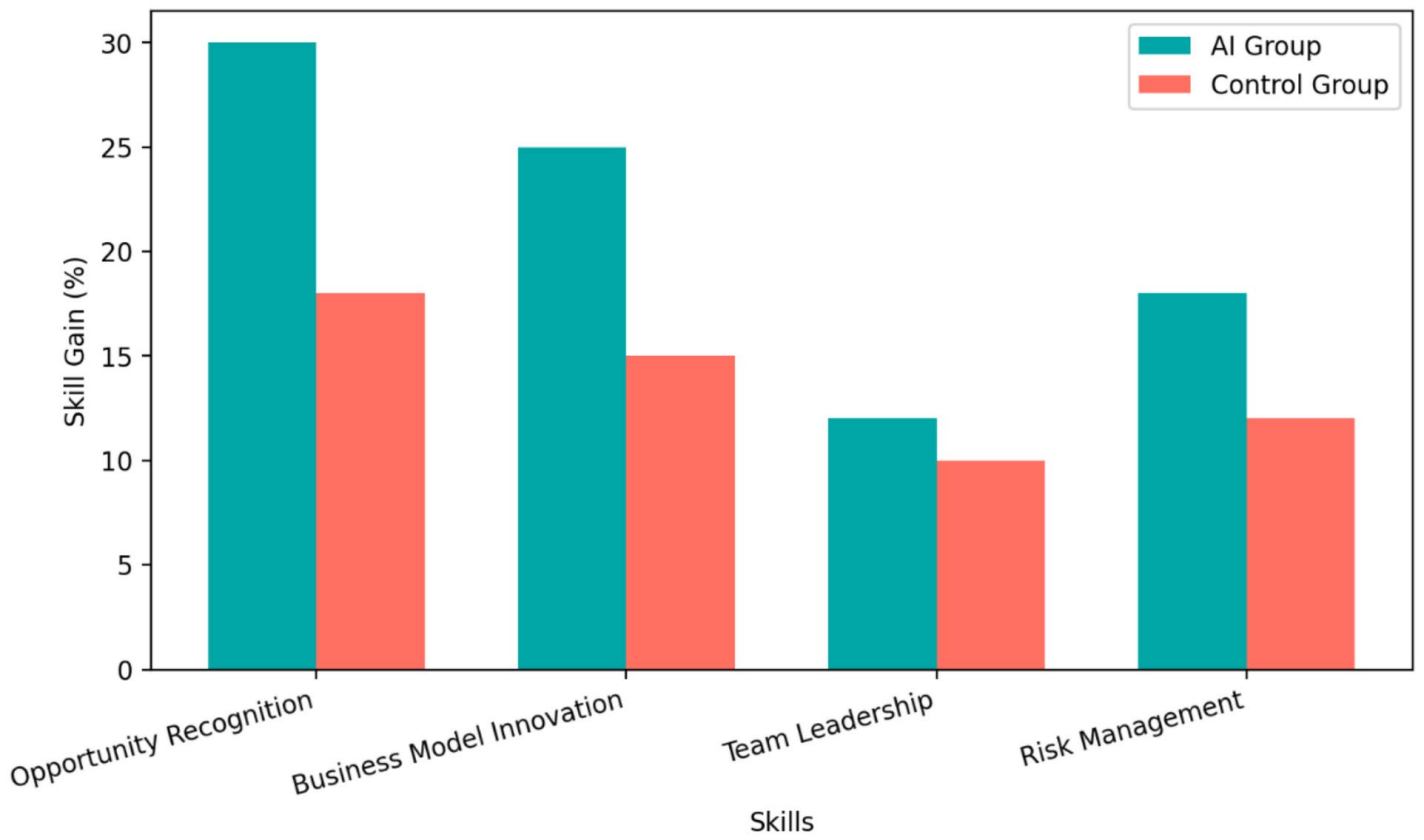
Self-reported gains (%) in opportunity recognition and business model innovation by instructional condition.

Students in the AI-enhanced group also reported stronger gains in entrepreneurial competence than students in the control group. Figure 3 shows that the AI-enhanced group reported a 30% improvement in opportunity recognition and a 25% improvement in business model innovation, with smaller gains observed in the control group.

These findings are important because entrepreneurship education aims not only to improve final products but also to strengthen learners’ perceived capability to identify opportunities and develop viable solutions. In this sense, AI-supported instruction appears to influence both performance and psychologically meaningful perceptions of competence.

### 3.3 Patterns of student engagement with AI tools

**Figure 4:**
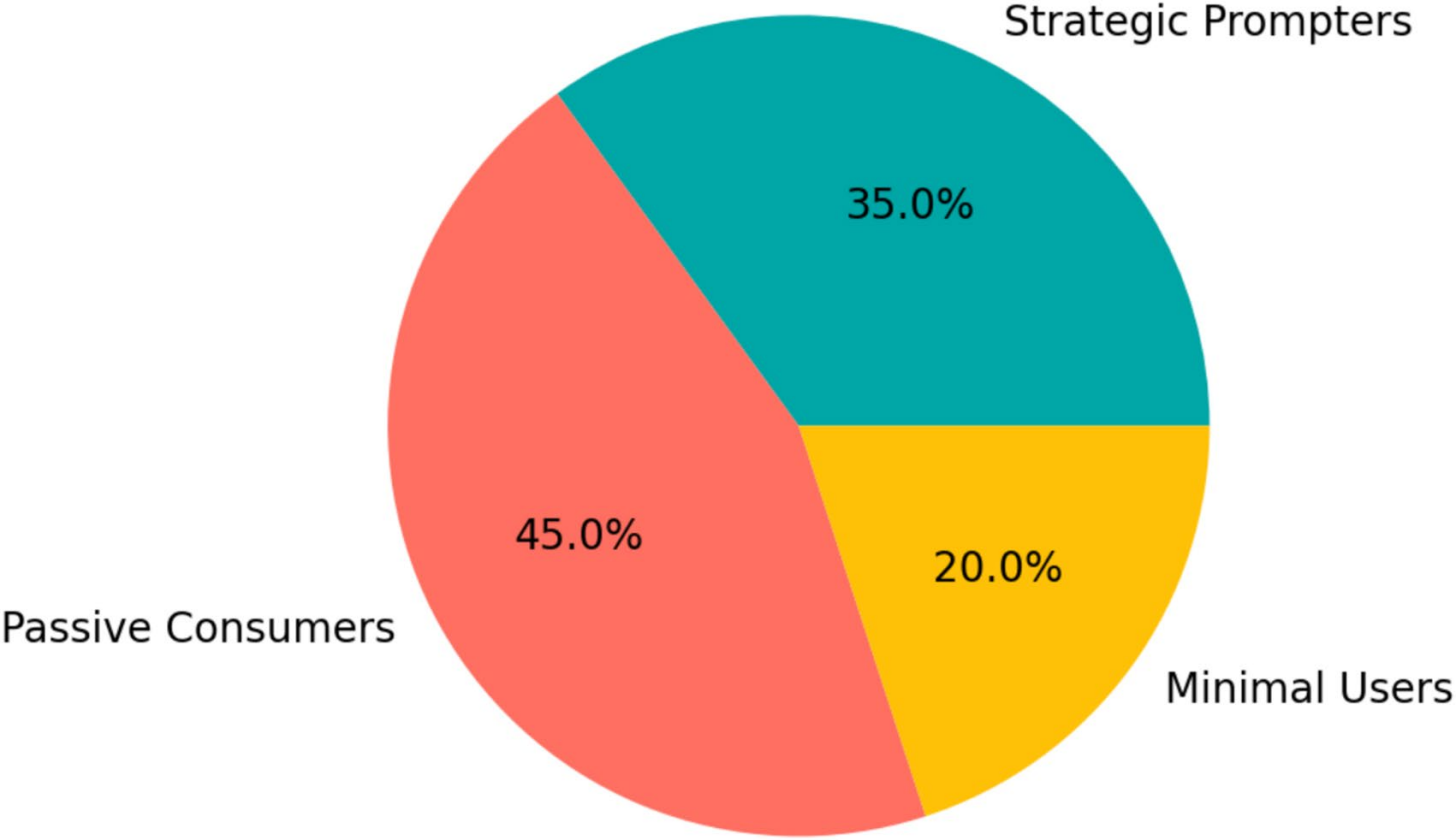
Distribution of student AI engagement typologies in the AI-enhanced group: strategic prompters, passive consumers, and minimal users.

Considerable heterogeneity emerged in how students used AI. As shown in Figure 4, 35% of students were categorized as Strategic Prompters, 45% as Passive Consumers, and 20% as Minimal Users. This distribution indicates that access to AI alone did not produce uniform learning behavior.

The distinction is theoretically meaningful. Strategic Prompters tended to use AI to explore alternatives, refine prompts, compare outputs, and support revision. Passive Consumers were more likely to accept generated content with limited evaluation, whereas Minimal Users engaged only weakly with the tools. These differences suggest that the educational value of AI is shaped by learner agency and self-regulation rather than by tool exposure alone.

### 3.4 Association between AI engagement and project outcomes

**Figure 5:**
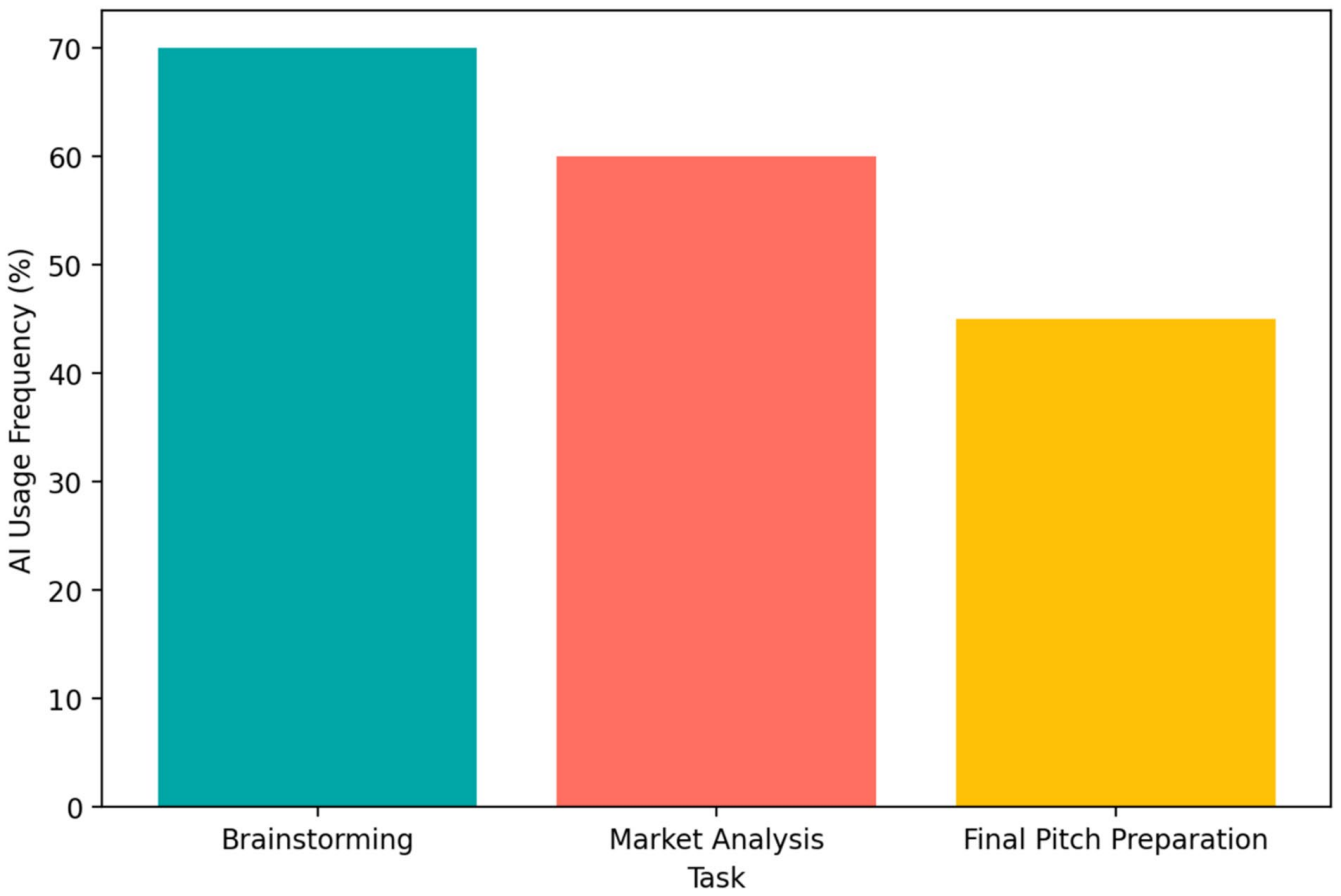
Frequency of AI use across entrepreneurship tasks, including brainstorming, market analysis, and pitch preparation.

**Figure 6:**
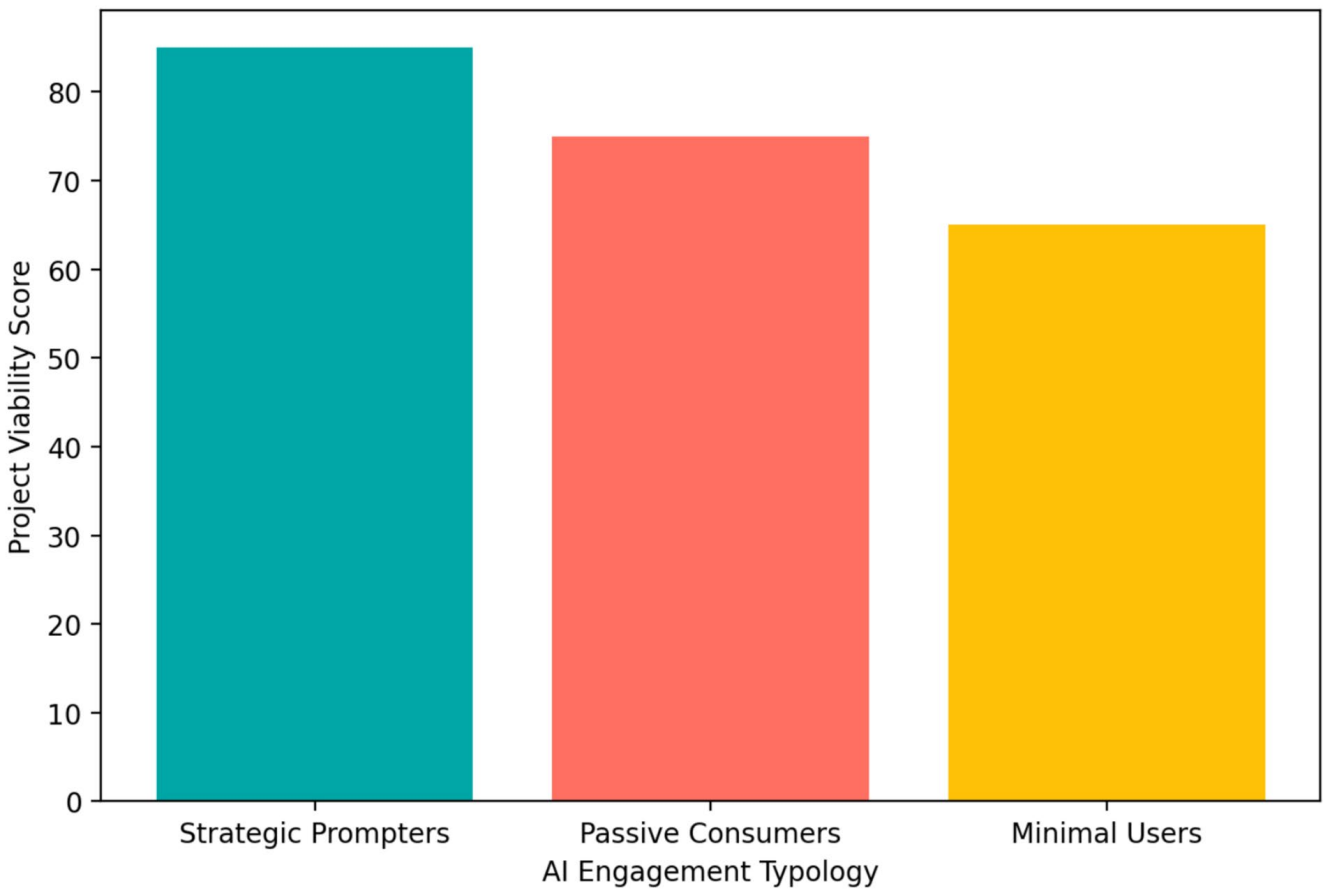
Mean project outcome scores by AI engagement typology.

Figure 5 indicates that brainstorming was the most frequent AI-supported task (70%), followed by market analysis (60%) and pitch preparation (45%). Figure 6 shows a clear gradient in project outcomes: Strategic Prompters achieved the highest average project score (85), followed by Passive Consumers (75) and Minimal Users (65).

Taken together, these findings suggest that the benefits of AI depend on the quality of engagement. Frequent AI use is not sufficient by itself; rather, stronger outcomes appear when students use AI in deliberate, evaluative, and revision-oriented ways. In psychological terms, this pattern is consistent with the view that high-quality learning depends on monitoring, reflection, and strategic control.

### 3.5 Moderating role of digital literacy

**Figure 7:**
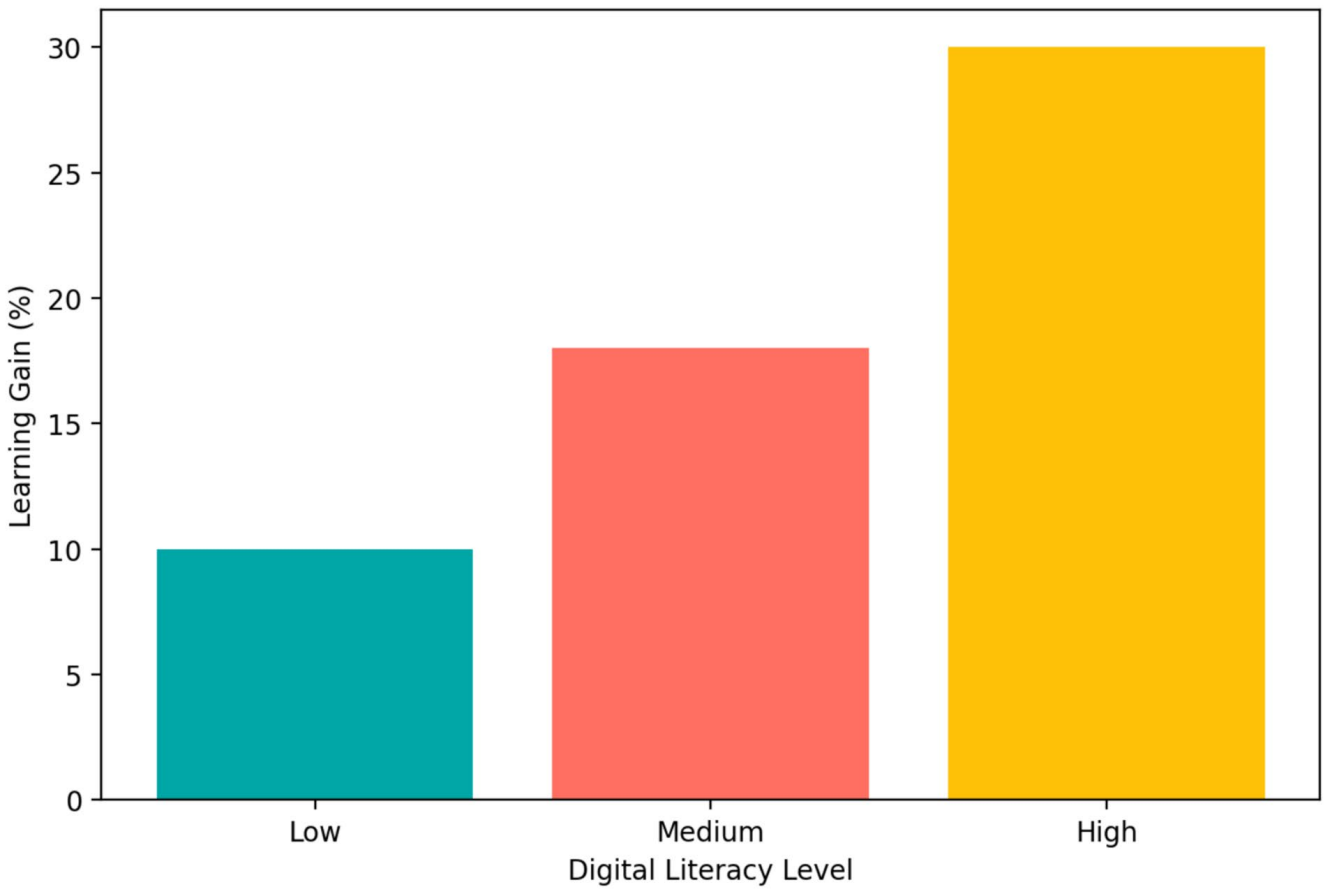
Digital literacy as a moderator of learning gains in AI-enhanced entrepreneurship education.

Digital literacy emerged as an important moderating factor. As shown in Figure 7, students with high digital literacy demonstrated the largest learning gains (30%), whereas students with medium and low digital literacy showed smaller gains (18 and 10%, respectively). This pattern was consistent with the hypothesized positive interaction between AI-enhanced instruction and students’ capacity to evaluate and integrate AI-generated information.

This finding indicates that students who were better able to evaluate and integrate AI-generated information benefited more from AI-enhanced instruction. It also suggests that AI-supported learning may widen existing disparities unless digital literacy is addressed as an explicit curricular objective rather than treated as a background skill.

### 3.6 Instructor preparedness and ethical concerns

**Figure 8:**
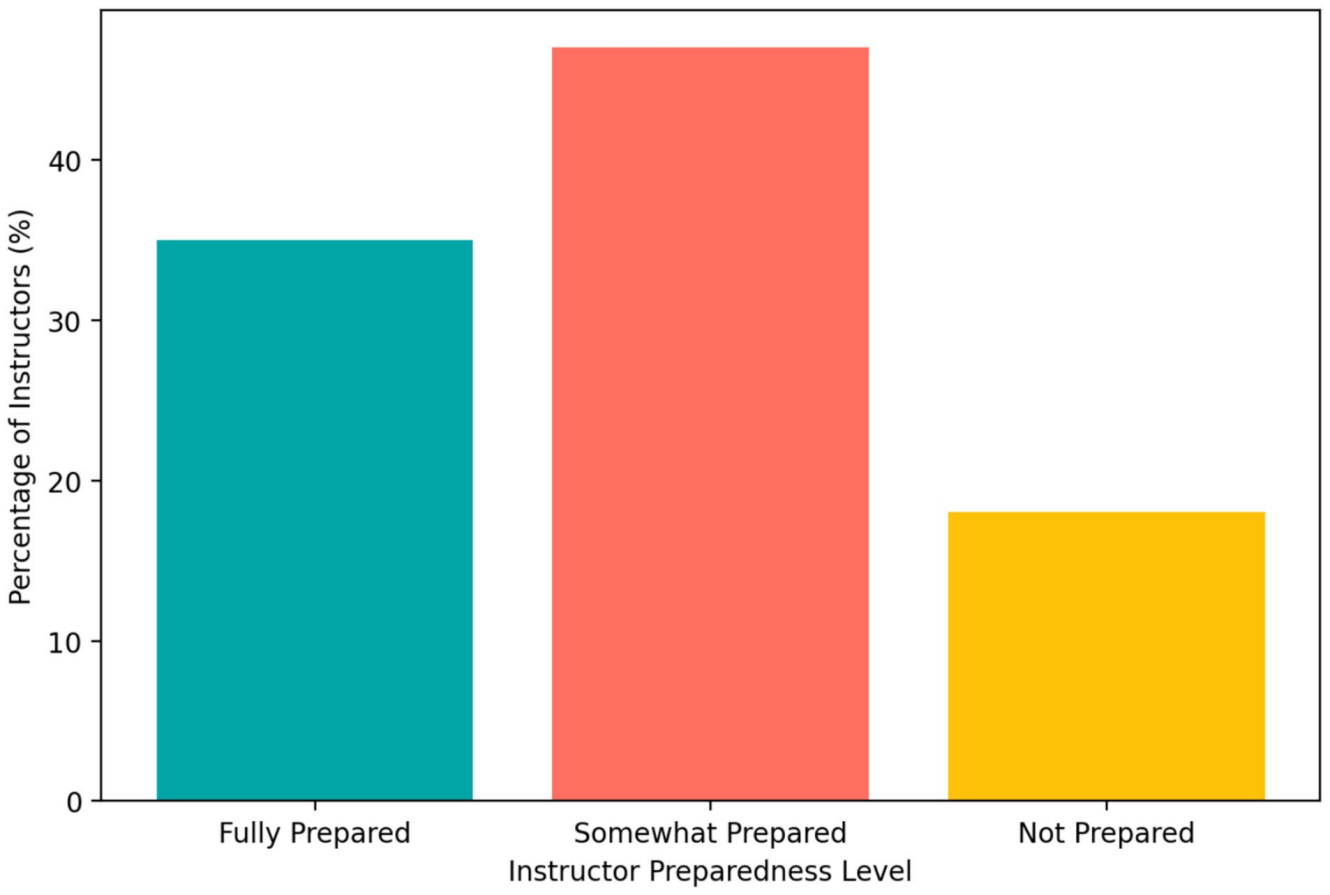
Instructor perceived preparedness to assess AI-assisted student work.

Instructor perceptions of preparedness to assess AI-assisted work were mixed. As shown in Figure 8, 35% reported feeling fully prepared, 47% somewhat prepared, and 18% unprepared. This pattern suggests that pedagogical adoption of AI may be proceeding faster than the development of shared assessment expertise.

**Figure 9:**
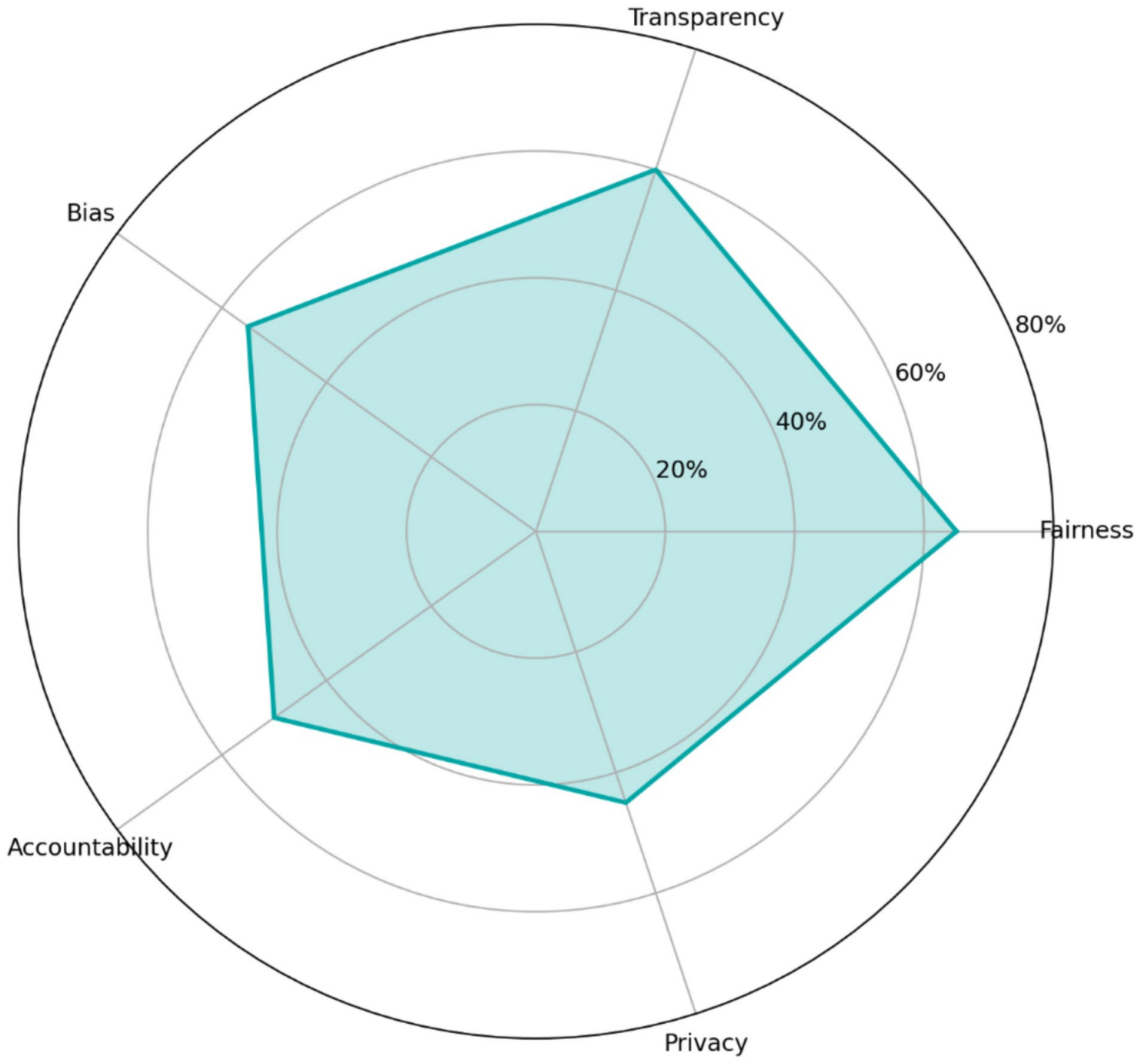
Instructor-reported ethical concerns related to AI use, including fairness, transparency, and bias.

Ethical concerns were also prominent. Figure 9 shows that 65% of instructors identified fairness as a major concern, followed by transparency (60%) and bias (55%). Qualitative responses indicated that teachers were especially concerned about distinguishing authentic student reasoning from AI-assisted production and about maintaining consistent standards across students with different levels of AI access and AI literacy.

### 3.7 Perceived equity effects across socio-economic status groups

**Figure 10:**
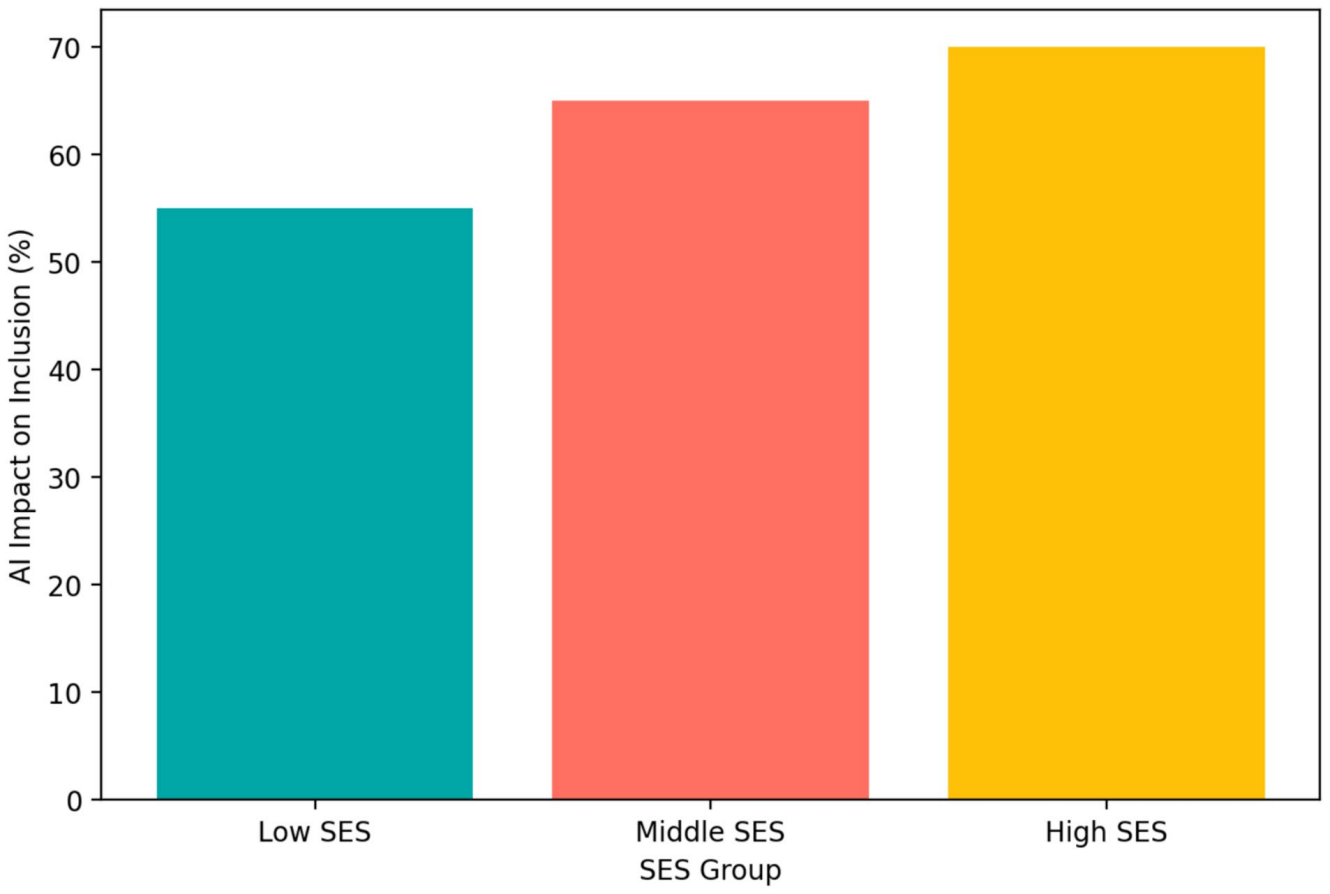
Perceived impact of AI-enhanced instruction on inclusion and equity across socio-economic status groups.

Students across socio-economic groups reported positive effects of AI-enhanced entrepreneurship instruction, but the magnitude of these perceived benefits varied. Figure 10 shows that students from higher socio-economic status groups reported the greatest positive impact on inclusion and equity (70%), followed by middle socio-economic status students (65%) and lower socio-economic status students (55%).

Although the findings do not suggest outright exclusion, they do indicate unequal benefit. From an educational psychology perspective, this matters because perceived equity can influence belonging, motivation, confidence, and willingness to participate in technology-mediated learning. The result implies that equitable AI integration requires more than access; it also requires differentiated support that helps diverse learners benefit from that access.

## 4 Discussion

The present study examined AI-enhanced entrepreneurship education in vocational colleges from an educational psychology perspective and found that AI-supported instruction was associated with stronger performance gains, greater self-reported entrepreneurial competence, and better project outcomes when students engaged with AI strategically. At the same time, the findings showed that these benefits were conditional rather than automatic. Engagement quality, digital literacy, and perceived fairness shaped whether and how AI contributed to learning.

First, the results support the view that AI can function as an instructional scaffold in practice-oriented entrepreneurship education. Students in the AI-enhanced condition improved more in business- plan performance and reported stronger gains in opportunity recognition and business-model innovation than students receiving comparable non-AI instruction. Analytically, this pattern suggests that AI was most useful not as a replacement for entrepreneurial thinking but as a structure for iterative practice, rapid comparison, and feedback-supported revision. This interpretation aligns with recent work arguing that generative AI is educationally valuable when embedded within guided self-regulated learning processes rather than treated as a shortcut to task completion (Kasneci et al., 2023; Farrokhnia et al., 2024; Lan and Zhou, 2025; Xia et al., 2026).

Second, the contrast among Strategic Prompters, Passive Consumers, and Minimal Users highlights the central importance of engagement quality. Theoretical discussions of AI in education sometimes treat tool exposure as though it were the operative variable. Our findings suggest a different conclusion: the same technology can support qualitatively different forms of learning depending on whether students interrogate, revise, and justify AI output. This moves the discussion from simple adoption to regulated use and strengthens the link between entrepreneurship education and self-regulated learning theory (Zimmerman, 2002; Panadero, 2017).

Third, digital literacy functioned as a meaningful moderating condition rather than as a background demographic feature. Students with stronger digital literacy benefited more from AI-enhanced learning, suggesting that they were better able to judge output quality, recognize limitations, and integrate AI suggestions into disciplinary reasoning. This result helps explain why equal access did not produce equal outcomes and supports current concerns that generative AI may reproduce or intensify digital inequalities unless institutions deliberately support learners in converting access into educational advantage (Beckman et al., 2025; Georgopoulou et al., 2025).

Fourth, fairness and assessment emerged as psychologically central rather than administratively secondary issues. Instructors reported substantial concern about fairness, transparency, and bias in evaluating AI-assisted student work, and students from lower socio-economic backgrounds reported smaller perceived equity gains. These findings indicate that AI-rich learning environments can become motivationally fragile when students are uncertain about authorship expectations, evaluation criteria, or the legitimacy of differential AI support. In this sense, perceived fairness should be treated as part of the learning ecology itself because it shapes trust, belonging, and willingness to engage.

Taken together, the results support a conditional model of AI-enhanced entrepreneurship learning. AI-supported instruction can be productive, but its value depends on instructional design, student regulation of tool use, sufficient digital literacy, and transparent assessment arrangements. This interpretation also clarifies why some discussion sections in the prior version risked sounding descriptive: the practical meaning of the results only becomes clear when the findings are interpreted through these interacting psychological processes.

### 4.1 Theoretical contribution

This study contributes to educational psychology by positioning AI-enhanced entrepreneurship education as a psychologically mediated learning ecology. Rather than assuming that AI is either beneficial or harmful in itself, the study suggests a conditional model in which outcomes depend on how students engage with AI, how well they regulate their learning, whether they possess sufficient digital literacy, and whether the instructional environment is experienced as fair and transparent. This framing connects entrepreneurship education with broader theories of self-regulated learning, student engagement, motivation, and feedback in educational contexts (Ryan and Deci, 2000; Zimmerman, 2002; Nicol and Macfarlane-Dick, 2006).

### 4.2 Practical implications

Several practical implications follow. First, vocational colleges should avoid treating AI as a stand-alone technological add-on. Instead, AI use should be embedded within explicit pedagogical routines that require students to question, compare, justify, and revise AI-generated outputs. Second, entrepreneurship curricula should include structured training in digital literacy and critical AI use, because students need support not only in operating tools but also in evaluating output quality, identifying limitations, and preserving authorship and judgment. Third, instructors need clearer and more transparent assessment frameworks for AI-assisted work. Rubrics should distinguish between the quality of final products and the quality of student reasoning, evidence use, and revision processes. Fourth, institutions should pay particular attention to equity-sensitive implementation. Students with lower prior digital experience may require more scaffolding, more guided practice, and more feedback if AI-supported learning is to be genuinely inclusive.

### 4.3 Limitations and future research

This study has several limitations. First, the quasi-experimental design supports comparison but does not provide the same level of causal inference as random assignment. Selection effects remain possible because intact classes were used, even though the study attempted to mitigate this threat through matched course conditions, a shared syllabus, common rubrics, and baseline comparability checks. Second, some measures relied on self-report and may therefore reflect subjective interpretation or social desirability. Third, the study focused on short-term instructional outcomes and did not examine whether the observed effects persisted over time.

Future research should employ longitudinal designs, finer-grained behavioral measures of AI interaction, and more explicit measurement of self-efficacy, autonomy, help seeking, motivation, and metacognitive monitoring. Additional work is also needed to identify which instructional designs help lower digital literacy students move from passive to strategic AI use and how fairness perceptions influence sustained engagement in AI-rich entrepreneurship classrooms.

## 5 Conclusion

AI-enhanced entrepreneurship education in vocational colleges shows clear promise, but its benefits are conditional rather than automatic. In the present study, students receiving AI-enhanced instruction demonstrated stronger gains in business plan performance and entrepreneurial competence than students receiving traditional instruction. However, these benefits depended on how students engaged with AI, how much digital literacy they possessed, and whether the learning environment supported transparent and fair assessment.

The findings suggest that AI should not be understood merely as a productivity tool in entrepreneurship classrooms. Instead, it should be viewed as part of a broader learning ecology that shapes cognition, motivation, self-regulation, and perceived equity. Effective implementation therefore requires psychologically informed instructional design, explicit support for critical AI use, and institutional attention to fairness and inclusion.

## Data Availability

The original contributions presented in the study are included in the article/supplementary material, further inquiries can be directed to the corresponding author.
